# High Selective Composite Polyalkylmethylsiloxane Membranes for Pervaporative Removal of MTBE from Water: Effect of Polymer Side-chain

**DOI:** 10.3390/polym12061213

**Published:** 2020-05-26

**Authors:** Ilya Borisov, Ivan Podtynnikov, Evgenia Grushevenko, Olga Scharova, Tatiana Anokhina, Sergey Makaev, Alexey Volkov, Vladimir Volkov

**Affiliations:** A.V. Topchiev Institute of Petrochemical Synthesis RAS, 119991 Moscow, Russia; podtynnikov@ips.ac.ru (I.P.); evgrushevenko@ips.ac.ru (E.G.); paker_avorash@list.ru (O.S.); tsanokhina@ips.ac.ru (T.A.); makaev@ips.ac.ru (S.M.); avolkov@ips.ac.ru (A.V.); vvvolkov@ips.ac.ru (V.V.)

**Keywords:** polyalkylmethylsiloxane, polymer side-chain, composite membranes, high selectivity, pervaporation, MTBE, water treatment

## Abstract

For the first time, the effect of the side-chain in polyalkylmethylsiloxane towards pervaporative removal of methyl tert-butyl ether (MTBE) from water was studied. The noticeable enhancement of separation factor during the pervaporation of 1 wt.% MTBE solution in water through the dense film (40–50 µm) can be achieved by substitution of a methyl group (separation factor 111) for heptyl (161), octyl (169) or decyl (180) one in polyalkylmethylsiloxane. Composite membrane with the selective layer (~8 µm) made of polydecylmethylsiloxane (M10) on top of microfiltration support (MFFK membrane) demonstrated MTBE/water separation factor of 310, which was 72% greater than for the dense film (180). A high separation factor together with an overall flux of 0.82 kg·m^−2^·h^−1^ allowed this M10/MFFK composite membrane to outperform the commercial composite membranes. The analysis of the concentration polarization modulus and the boundary layer thickness revealed that the feed flow velocity should be gradually increased from 5 cm·s^−1^ for an initial solution (1 wt.% of MTBE in water) to 13 cm·s^−1^ for a depleted solution (0.2 wt.% of MTBE in water) to overcome the concentration polarization phenomena in case of composite membrane M10/MFFK (T_exp_ = 50 °C).

## 1. Introduction

Methyl tert-butyl ether (MTBE) is used as an additive to increase the octane number of gasoline, which allows to improve the antiknock properties of the fuel and to reduce the concentration of CO in exhaust gases as well as the cost of gasoline production [1,2]. However, as a result of leakage during storage and transportation of both MTBE and gasoline, there is a serious threat of groundwater pollution [3,4,5]. For instance, MTBE is the second most frequently detected compound in coastal and groundwater [6]. MTBE has a negative effect on the taste of water, irritates the skin and eyes of a person, is able to suppress the nervous system and can stimulate various damages in DNA [7,8]. Due to the problem of groundwater pollution with MTBE, its use was limited in the European Union and almost completely prohibited in the USA and Canada in the 2000s. Despite these limitations, the global production of MTBE was about 12 million tons per year in 2011 [9].

A lot of research has been devoted to the problem of MTBE removal from wastewater [6,10,11,12,13,14,15,16,17]. Such methods as adsorption, air purging, chemical oxidation, and biological treatment are considered for treating water contaminated with MTBE [11]. To date, biological treatment is the most common method of cleaning wastewaters containing MTBE [15,16]. The use of microorganisms to convert organic pollutants to carbon dioxide and water allows the achievement of a high degree of wastewater treatment. Nevertheless, microorganisms are highly sensitive to environmental conditions (temperature, runoff composition, pH, biochemical oxygen demand, etc.), therefore the efficiency of the cleaning process is highly dependent on its conditions [17]. The biomass leak from reactors with a stream of purified water requires additional purification of water from microorganisms. All these factors dictate the need to find alternative methods of cleaning water from MTBE.

A promising method for water treatment is membrane separation, in particular, the pervaporation process [18]. The advantages of this method include the absence of reagents, continuous and mild conditions of the separation process [19]. Pervaporation has been extensively studied for the separation of oxygenates from aqueous media [17,18,19,20,21,22,23,24,25,26,27,28,29,30]. It has been shown that pervaporation has great potential for the removal of MTBE from groundwater and drinking water both at the laboratory and pilot levels [31]. However, it was found that the main problem in the removal of MTBE from water by pervaporation is the low mass transfer rates (permeability) of industrial membranes. At the same time, the authors note the need to minimize the water flux through the membrane. An increase in water flux leads to a significant increase in costs associated with vacuum pump operation and condensation of permeate [31]. Thus, membranes with high MTBE permeance and low water ones are required.

Existing industrial plants use polysiloxane-based membranes [32] to extract volatile organic compounds from water solutions. Membranes based on silicone rubbers are widely used because they are easy to manufacture, chemically resistant and have stable transport characteristics. Ceramic hydrophobic membranes are also promising for MTBE removal, but they were manufactured and studied only at the laboratory level [33,34]. The most common material for hydrophobic pervaporation is polydimethylsiloxane (PDMS). It has the highest permeability among siloxane rubbers, but its selectivity for organic components is not sufficient. This is due to the high water permeability coefficient of PDMS (40,000 Barrer) [32,35].

The increase in the selectivity of silicone rubbers could be achieved by introducing hydrophobic zeolites into the polymer matrix [36], co-polymerization of dimethylsiloxane with more hydrophobic monomers [37], as well as the introduction of hydrophobic substituents into the main and side-chain of the polymer [23,38,39,40]. For example, polyheptylmethylsiloxane demonstrates a significantly higher n-butanol-water separation factor (97) compared to PDMS (38) [38]. Consequently, the development of highly permeable membranes based on polysiloxanes with increased hydrophobicity is a promising direction for solving the problem of removal MTBE from water.

However, the use of membranes with increased selectivity and permeability for the separation of MTBE from dilute solutions is inevitably accompanied by an increase in the negative contribution of the “concentration polarization” effect, i.e., a decrease in the driving force of the separation process due to a decrease in the concentration of the selectively permeating component (MTBE) in the boundary layer near the membrane surface [41,42,43]. Assessing the effect of concentration polarization plays an important role in optimizing the design of the module and conditions of the pervaporation process [44].

Highly permeable pervaporation membranes based on polyalkylmethylsiloxanes with increased hydrophobicity were developed and studied in this work in order to solve the problem of efficient removal of MTBE from water. The effect of concentration polarization on the permeate flux and the MTBE/water separation factor was studied using different pervaporation process parameters (feed flow rate, feed temperature and composition).

## 2. Materials and Methods

### 2.1. Materials

The synthesis of polyalkylmethylsiloxanes was performed using polymethylhydrosiloxane (PMHS) with number-average molecular weight Mn 1700–3200 g∙mol^−1^ (Sigma-Aldrich, Saint Louis, MO, USA), a Karstedt’s catalyst (a platinum complex of 1,3-divinyl-1,1,3,3-tetramethyldisiloxane in xylene, Sigma-Aldrich, Saint Louis, MO, USA), 1-heptene (97%, Sigma-Aldrich), 1-octene (98%, Sigma-Aldrich, Saint Louis, MO, USA) 1-decene (94%, Sigma-Aldrich, Saint Louis, MO, USA), 1,7-octadiene (98%, Sigma-Aldrich, Saint Louis, MO, USA), *n*-hexane (99%, Chimmed, Podol’sk, Russia) without further purification.

A microfiltration membrane MFFK-1 (Vladipor, Vladimir, Russia) was used as membrane support. It was made of a porous layer of fluoroplastic F42L, coated on a nonwoven fabric from polyethylenterephthalate.

### 2.2. Synthesis of Polyorganosiloxanes and Preparation of Dense Membranes

A membrane-based on polydimethylsiloxane (PDMS) was used as a reference. To produce its casting solution, vinyl-terminated PDMS was crosslinked with PMHS by hydrosilylation in the presence of a Karstedt’s catalyst. The ratio of PDMS:PMHS:catalyst was 10:1:0.01.

**Figure 1:**
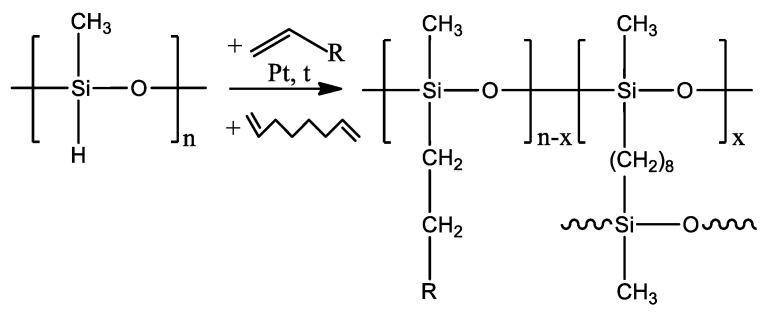
Reaction scheme for the production of polymethylsiloxanes with substituted side-chain.

Polymethylsiloxanes with substituted side-chain were synthesized by the hydrosilylation reaction according to the previously proposed procedure [45]. Membranes were synthesized in a one-stage process, which was chosen because of the possibility of using the reaction mixture as a casting solution, the need of just one catalyst (Karstedt’s catalyst), and the ease of varying the nature of the side substituent and the crosslinking agent. The reaction scheme is shown in Figure 1.

In accordance with this method, 3 wt.% solution of polymethylhydrosiloxane (PMHS) in 1-hexane were mixed with 1-alkene (modifier), after which Karstedt’s catalyst was added. The resulting mixture was stirred for 2 h at 60 °C under Liebig condenser. After that, a crosslinking agent, 1,7-octadiene, was added to the solution with the mole ratio of the crosslinking agent to the modifier 5/95. The resulting mixture was used as a casting solution.

Films from polyorganosiloxanes were produced by casting a polymer solution onto a stainless steel mesh (mesh size 40 μm) fixed on a Teflon surface, followed by drying to constant weight at a temperature of 60 °C (24 h was enough). The thickness of the produced membranes (δ [µm]) was calculated by using the geometric dimensions of the steel mesh and the difference in the mass of the mesh and the resulting membrane (Equation (1)):

(1) $$\delta =\frac{4\left(m_{2}-m_{1}\right)}{\pi d^{2}\rho }$$

where *m*_1_ is the steel mesh mass [g], *m*_2_ is the weight of the membrane on the steel mesh [g], *d* is the membrane diameter [cm], *ρ* is the membrane density [g cm^−3^]. The film thickness varied in the range of 40–50 microns. Short names of the prepared membranes and the glass transition temperature (measured by calorimetry) of the synthesized polymers are presented in Table 1.

### 2.3. Composite Membrane Preparation

Composite membranes were prepared by the kiss coating method, which involves pulling a porous support over the surface of the casting solution so that a meniscus forms, which prevents the support from immersion in the casting solution [46]. The microfiltration membranes MFFK-1 (Vladipor, Vladimir, Russia) were used as a support for flat composite membranes. The porous support was impregnated with water before applying the selective layer. After that, the composite membranes were dried on a heating table at 60 °C for 2 h. Finally, the membranes were dried under vacuum at 60 °C for 18 h.

### 2.4. Differential Scanning Calorimetry

Calorimetric studies were performed in argon with calorimeter Mettler DSC823 (Mettler-Toledo GmbH, Columbus, OH, USA) at a scanning rate of 10 deg∙min^−1^ in the range 140–100 °C. The glass transition can be observed as the midpoint of a step in the baseline of the measurement DSC curve (Figure S1).

### 2.5. Vacuum Pervaporation

**Figure 2:**
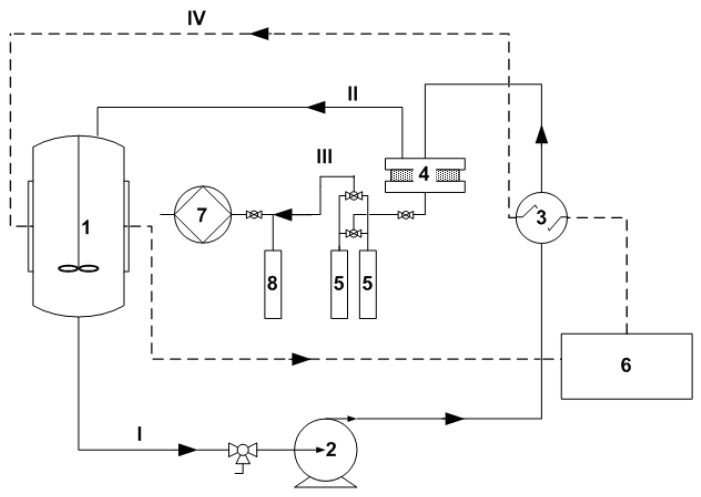
Layout of the vacuum pervaporation setup.

Pervaporation experiments were carried out on the setup shown in Figure 2.

The initial feed mixture of water and MTBE was poured into a 1 L container (1). The mixture was heated in the heat exchanger (3) and fed into the membrane module (4) in a circulating mode using an Ismatec gear pump (Ismatec, Wertheim, Germany) (2). The volumetric flow rate of the feed mixture varied from 20 to 220 mL min^−1^ (linear velocity 0.5–5.2 cm s^−1^). The effective membrane area was 13.9 cm^2^. Permeate vapors were condensed in glass traps placed in Dewar vessels with liquid nitrogen (−196 °C) (5). The continuous operation of the installation throughout the experiment was ensured by the presence of two traps, working in parallel. A safety trap (8) was used to prevent permeate vapor from entering the vacuum pump (7). The vacuum pump was turned off at the end of the experiments, and the trap (8) was defrosted. The completeness of condensation of permeate vapors in the traps (5) during the experiments was confirmed by the absence of liquid traces in the trap (8). The temperature of the feed mixture was maintained with an accuracy of ± 0.1 °C using a LOIP LT-100 liquid thermostat (LOIP, Saint-Petersburg, Russia) (6). The driving force of the mass transfer process (a pressure of ~0.05 mbar in the submembrane space) was created by and maintained with an Ebara PDV-250 vacuum pump (EBARA, Tokyo, Japan) (7).

The study of pervaporation of 0.2–1.0 wt.% MTBE-water solutions was carried out at a temperature of 30–60 (±0.1) °C. The initial solution was prepared from MTBE (laboratory grade, chemically pure) and distilled water by the gravimetric method. The exact concentrations of MTBE in the feed mixture and permeate were determined by gas chromatography on a Kristallux-4000M gas chromatograph (Meta-chrome, Yoshkar-Ola, Russia) equipped with a thermal conductivity detector. Chromatography parameters: evaporator temperature −230 °C, column temperature −180 °C, detector temperature −230 °C. The measurements were carried out using a 1 m long column, filled with a Porapak Q sorbent.

### 2.6. Process Parameters Calculation

Flux was estimated by weighting permeate collected over a given period of time. Total permeate flux *J_t_* [kg m^−2^ h^−1^] was calculated as:

(2) $$J_{t}{}^{{}_{}}=\frac{\Delta m}{S\cdot \Delta t}$$

where Δ*m* is the weight of the permeate [kg], which passed through the membrane with area *S* [m^2^] within a time Δ*t* [h].

Total permeate volume flux (*J_v_*) [m^3^ m^−2^ h^−1^] was calculated using Equation (3):

(3) $$J_{v}=\frac{J^{W}}{\rho^{W}}+\frac{J^{M}}{\rho^{M}}=\sum \frac{J^{i}}{\rho^{i}}.$$

There, mass fluxes of MTBE (*J^M^*) and water (*J^W^*) in the permeate were:

(4) $$J^{M}=J_{t}\cdot C_{wp}^{M}$$

(5) $$J^{W}=J_{t}-J_{M}$$

where $C_{wp}^{M}$ is the mass fraction of MTBE in permeate, *ρ^M^* and *ρ^W^* are the densities of MTBE and water [kg m^−3^] at 20 °C.

Feed velocity in the flow channel along the membrane surface [cm s^−1^] was calculated as:

(6) $$v_{f}=\frac{V_{f}}{H\cdot L}$$

*V_f_* is the feed flow rate [cm^3^ s^−1^], *H* is the fluid channel height [cm], *L* is the channel width [cm].

Apparent enrichment factor (*E*) and apparent separation factor (*α*) were determined using Equations (6) and (7).

(7) $$E=\frac{C_{mp}^{M}}{C_{mf}^{M}}$$

(8) $$\alpha =\frac{C_{wp}^{M}\cdot C_{wf}^{W}}{C_{wp}^{W}\cdot C_{wf}^{M}}$$

where $C_{mp}^{M}$ and $C_{mf}^{M}$ are the molar fraction of MTBE in feed and in permeate [mol mol^−1^], $C_{wp}^{M}$ and $C_{wp}^{W}$ are the mass fraction of MTBE and water in permeate, $C_{wf}^{M}$ and $C_{wf}^{W}$ are the mass fraction of MTBE and water in feed [g g^−1^].

Phase transition separation factor (*α_v_*) was determined using Equation (9).

(9) $$\alpha_{v}=\frac{C_{wv}^{M}\cdot C_{wf}^{W}}{C_{wv}^{W}\cdot C_{wf}^{M}}$$

where $C_{wv}^{M}$ and $C_{wv}^{W}$ are the mass fraction of MTBE and water in saturated vapor above the feed.

Membrane selectivity (*α_m_*) was determined using Equation (10).

(10) $$\alpha_{m}=\frac{\alpha }{\alpha_{v}}$$

Permeability [mol m m^−2^ Pa^−1^ h^−1^] was calculated as:

(11) $$P^{i}=\frac{J^{i}\cdot l}{M^{i}\cdot \Delta p^{i}}$$

where *l* is the membrane thickness [m], *M^i^* is the molar mass of component *i* [kg mol^−1^], Δ*p^i^* is the trans-membrane pressure difference of component *i* [Pa] (calculated from Henry’s law constants obtained from Reference [44]).

Relative permeability [-] was found as:

(12) $$P_{r}^{i}=\frac{P^{i}}{P_{PDMS}^{i}}$$

where $P_{PDMS}^{i}$ is the permeability [mol m m^−2^ Pa^−1^ h^−1^] of component *i* through PDMS.

Molar concentrations of MTBE in feed ($C_{mf}^{M}$) or permeate ($C_{mp}^{M}$) can be calculated with the following formula:

(13) $$C_{m}^{M}=\frac{C_{w}^{M}\cdot M^{W}}{C_{w}^{M}\cdot M^{W}+\left(1-C_{w}^{M}\right)M^{M}}$$

derived from the definition of $C_{w}^{M}$, which is the mass fraction of MTBE (Equation (14)).

(14) $$C_{w}^{M}=\frac{m^{M}}{m^{M}+m^{W}}=\frac{C_{m}^{M}\cdot M^{M}}{C_{m}^{M}\cdot M^{M}+\left(1-C_{m}^{M}\right)M^{W}}$$

where *M^M^* and *M^W^* are the molar masses of MTBE and water, respectively [kg mol^−1^].

Intrinsic enrichment factor [-] *E*_0_ was calculated by the method described in Reference [43].

Intrinsic separation factor [-] *α*_0_ was calculated using Equations (7), (8), (13) and (14).

To describe the extent of concentration polarization, so-called concentration polarization modulus (*C_mm_/C_mf_*) can be determined.

(15) $$\frac{C_{mm}^{M}}{C_{mf}^{M}}=\frac{E}{E_{0}}$$

where *C_mm_* is the molar fraction of the substance at the surface of the membrane.

Concentration polarization modulus was calculated according to the Equation (15), using the values of *E*_0_.

Subsequently, boundary layer thickness δ [m] was calculated [42]:

(16) $$\delta =\frac{D}{J_{v}}\ln\left(\frac{1/E_{0}-1}{1/E-1}\right)$$

Knowing the water viscosity and the MTBE molar volume, and using the empirical formula (Equation (17)), value of diffusion coefficient [m^2^ s^−1^] for the temperature 50 °C was estimated. Calculations of MTBE diffusion coefficients at a given constant temperature were performed using a method developed in Reference [47].

(17) $$D=0.36\cdot \frac{13.26\cdot 10^{-5}V_{M}^{0.599}}{h^{1.14}}$$

where h is the water viscosity [Pa s], $V_{M}^{}$ is the MTBE molar volume [cm^3^ mol^−1^].

## 3. Results and Discussion

### 3.1. Effect of Side-Chain Length on the Pervaporation Characteristics of Polyalkylmethylsiloxanes

Table 2 lists some physicochemical properties of components of the feed mixture (water and MTBE) and polyalkylmethylsiloxanes (M1, M7, M8 and M10) used in this study. As can be seen, the kinetic diameter of MTBE molecules is larger than for water by a factor of 2.5, and, consequently, the diffusion selectivity of MTBE/water shall be lower than 1. However, the polyalkylmethylsiloxanes demonstrate greater affinity to organic components over the water molecules and showed high selectivity in pervaporative recovery of organics from aqueous solutions since the sorption selectivity plays a major role in the overall selectivity of pervaporation process [38,39,40]. Such affinity between the polymer and water or MTBE was evaluated by using the distance parameters calculated from the Hansen solubility group parameters (δ) [48]. The smaller the value of the distance parameter, the greater the sorption affinity can be expected between the polymer and corresponded solvent. Thus, it can be concluded that the replacement of the methyl group with a heptyl, octyl or decyl one made the polymeric material less favorable for water due to increasing the distance parameter for the water-polymer from 36.6 up to 40.6 MPa^1/2^ (see Table 2). At the same time, the polymers with long side alkyl chain became more favorable for the comparison of MTBE with PDMS because of the drop of the distance parameter for the MTBE-polymer from 3.6 down to 2.6 MPa^1/2^. Therefore, a higher solubility selectivity of MTBE/water and, consequently, the permeability selectivity can be expected for polyalkylmethylsiloxanes compared to PDMS.

**Figure 3:**
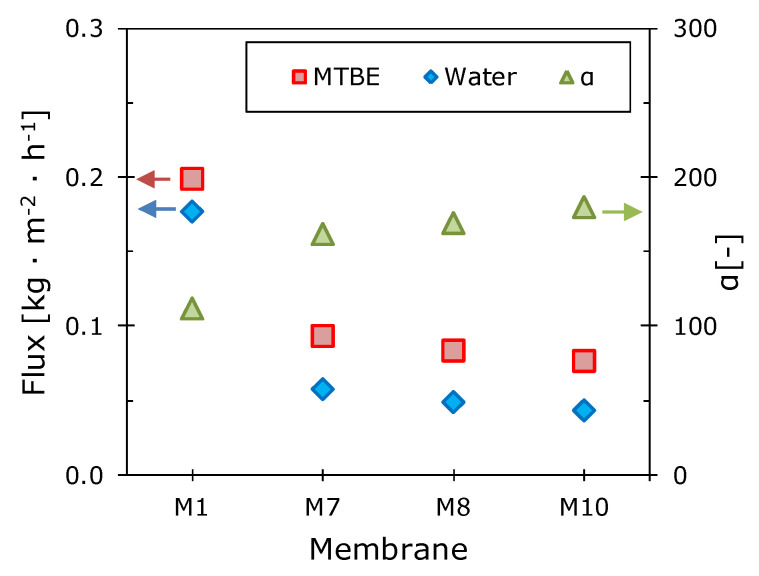
The dependences of the partial fluxes of MTBE and water and the MTBE/water separation factor on the side-chain length for the studied polyalkylmethylsiloxane membranes. The membrane’s thickness was 50 microns. T = 30 °C, 1% wt. MTBE in water.

The pervaporation characteristics of the membranes were studied by separation of a 1 wt.% MTBE solution in water by vacuum pervaporation at a temperature of 30 °C. The partial fluxes of MTBE and water, as well as the separation factor MTBE/water, are presented in Figure 3.

**Figure 4:**
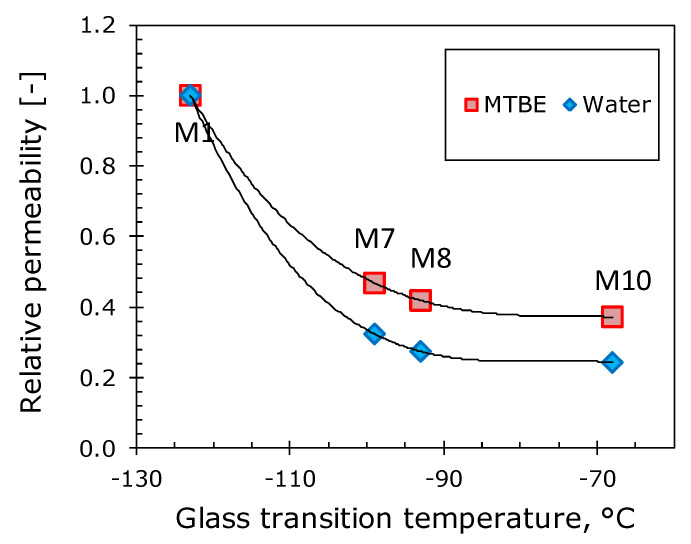
The dependences of the relative permeabilities of MTBE and water in polyalkylmethylsiloxanes on their glass transition temperature (relative to the corresponding values for M1).

It is evident that with an increase in the length of the side-chain substituent, the partial fluxes of the components decrease, while the separation factor increases from ~110 to 180 for M10. To explain these phenomena, Figure 4 shows the dependences of the reduced pervaporation parameters (relative to the corresponding values for M1 membrane) on the glass transition temperature of the investigated polymers. Membrane permeability reduces several times with an increase in the glass transition temperature of the polymer. The MTBE permeability coefficient in going from M1 to M10 gradually decreases by 62%, while in water it decreases by 76%. This leads to a gain in the selectivity of the polymer, due to an increase in its hydrophobicity.

The separation of liquids by pervaporation on the molecular level is described by the “solution-diffusion” model. The transport of low molecular weight substances in elastomers is carried out via the elements of free volume formed as a result of segmental mobility in the polymer. The higher the molecular mobility, the higher the fraction of elastomer free volume and vice versa. The experimental temperature was the same for all polymers (30 °C), therefore, the higher the glass transition temperature of the investigated polymer, the lower its free volume at 30 °C and, consequently, the fluxes of the separated components should also be lower. This explains the downward trend in the partial fluxes of MTBE and water (Figure 4) as the length of the side-chain substituent increases and, accordingly, the glass transition temperature of the polymer increases. A decrease in permeability coefficients with an increase in the glass transition temperature of elastomers has previously been repeatedly noted in the literature [49,50,51].

It should be emphasized that dense membranes with a thickness of tens of microns do not meet the requirements of industrial pervaporation modules [32]. High mass transfer coefficients necessary for the efficient separation of MTBE from water can be achieved by reducing the thickness of the selective layer and creating highly permeable composite membranes. For this purpose the selectivity of the material is crucial. Reducing the water flux will significantly reduce the costs associated with vacuum pumps operation and condensation of permeate [32]. Dense M10 membrane showed the highest selectivity for MTBE; therefore, the M10/MFFK composite membrane was prepared with this polymer.

### 3.2. Pervaporation Characteristics of the Composite Membrane M10/MFFK for MTBE Removal

**Figure 5:**
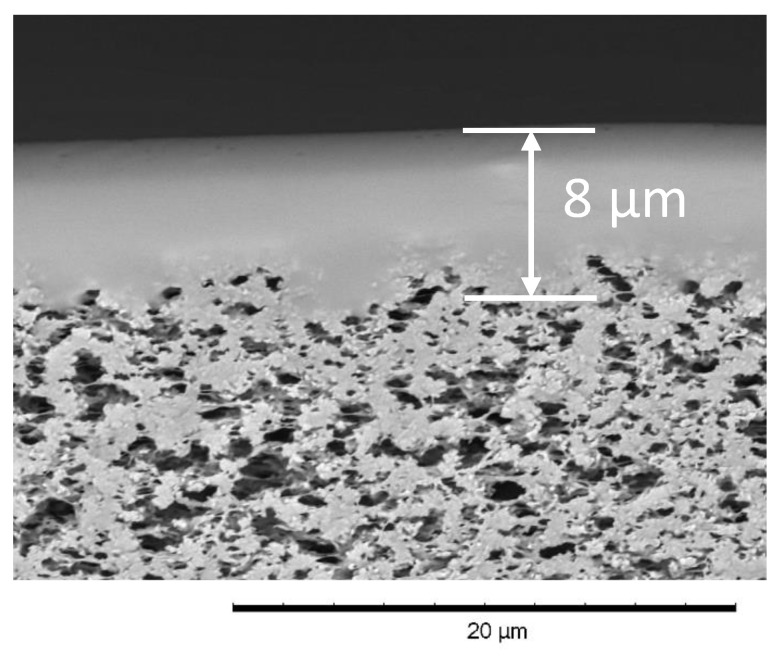
SEM image of the cross-section of the composite membrane M10/MFFK.

A micrograph of the cross-section of the composite membrane M10/MFFK is presented in Figure 5. The estimated thickness of the selective layer from M10 polymer material is 6–8 μm and polydecylmethylsiloxane penetrates into the pores of the support to a small depth of about 1–2 μm.

Table 3 compares the pervaporation characteristics of the M10/MFFK membrane with the literature data for composite membranes used for the removal of MTBE from water. The M10/MFFK composite membrane has high values of permeate flux (0.82 kg m^−2^ h^−1^) and separation factor (310) that exceed the characteristics of the best commercial composite membranes. Moreover, the MTBE/water separation factor of the composite membrane M10/MFFK (310) is significantly higher than the value for dense M10 membranes with a thickness of 50 μm (180, Figure 3). Such findings revealed high potentials of composite-type membranes for MTBE/water separation. It was argued that such an increase in selectivity can be caused by the partial intrusion of the polymer of the selective layer into the upper layer of the porous support (Figure 5), which prevents the siloxanes from swelling and reduces the water flux. A similar effect was observed in Reference [52], where the separation selectivity of the binary n-butane/methane mixture increased from 10 to 12 for the POMS-based composite membrane because of the partial intrusion of the polymer into the pores of the support.

The increased selectivity of composite membranes compared to the dense films made of the same material can be also explained by the contribution of porous support in the pervaporation process. For instance, it was reported earlier [53] that composite polyphenylene oxide (PPO) membranes on MFFK have a higher selectivity for methyl acetate/water separation compared with PPO films. The authors explained this effect by the increased solubility of methyl acetate in the material of the porous support made of fluoroplastic F42L. This led to increased transport of the organic component through the membrane resulted in higher selectivity. A similar effect could be expected in the case of the separation of MTBE/water mixture by using the composite membrane based on MFFK support.

**Figure 6:**
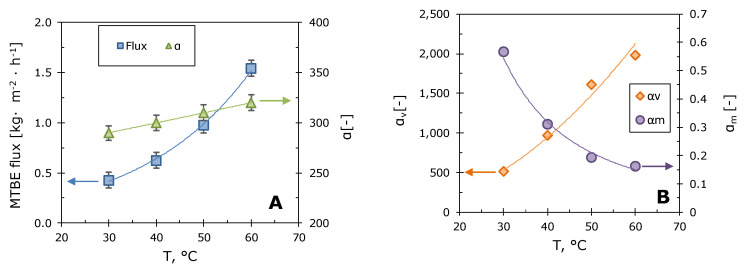
Temperature dependences of MTBE flux and MTBE/water separation factor (**A**); temperature dependences of the phase transition separation factor *α_v_* and membrane selectivity *α_m_* (**B**). The linear velocity of the feed mixture was 4.0 cm·s^−1^, 1 wt.%. MTBE in water, composite membrane M10/MFFK.

In order to determine the effect of the pervaporation process parameters on the transport and separation characteristics of the M10/MFFK membrane, the separation temperature, the concentration of MTBE in the water solution, and the feed flow rate in the membrane module were varied. Figure 6A shows the dependences of the permeate flux and the MTBE/water separation factor in the temperature range 30–60 °C.

It can be seen that with the increase in temperature, the permeate flux and the separation factor gradually grow. The increase in permeate flux is caused by an increase in diffusion coefficients with temperature in accordance with the Arrhenius law. This effect was observed in Reference [56], where the influence of temperature on the pervaporation of aromatic compounds using PDMS and POMS membranes was studied. The authors [31] also observed an increase in MTBE mass transfer coefficients across polysiloxane membranes with increasing temperature.

In order to understand the role of the membrane in the pervaporation of MTBE–water solutions, the components of the separation factor were found: phase transition separation factor (*α_v_*) and membrane selectivity (*α_m_*) [57,58]. The dependences of these values on the separation temperature are shown in Figure 6B. An increase in the total separation factor with temperature is clearly associated with an increase in the phase transition separation factor. A similar result has been demonstrated earlier in References [57,59].

A sharp four-fold drop in membrane selectivity with increasing separation temperature is unusual (Figure 6B). For example, authors [57] observed an increase in membrane selectivity for butanol with increasing temperature. In our case the drop is most likely associated with the concentration polarization effect caused by high permeate fluxes and membrane selectivity. As a result, the concentration of MTBE at the membrane-liquid interface is reduced relative to the concentration in the bulk feed solution. This leads to a decrease in MTBE flux and apparent membrane selectivity. The impact of the linear velocity and concentration of the feed solution on the pervaporation process was used to assess the concentration polarization effect and calculate the real transport and separation characteristics of the membranes.

### 3.3. Concentration Polarization in the Pervaporative Separation of MTBE–Water Solutions

**Figure 7:**
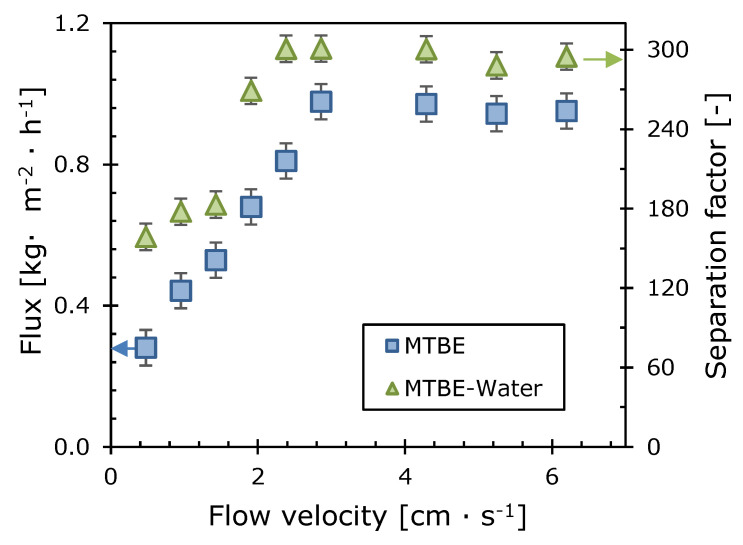
The dependences of the MTBE flux and the MTBE/water separation factor on the feed linear velocity. T = 50 °C, 1% wt. MTBE in water, composite membrane M10/MFFK.

A temperature of 50 °C was chosen for concentration polarization experiments because of high fluxes and separation factors, and, at the same time, the experimental error associated with the volatility of MTBE is not high. The boiling point of MTBE is 55.2 °C; therefore, at 60 °C the concentration of its solutions drops very quickly due to evaporation. The feed flow velocity varied from 0.5–5.2 cm·s^−1^. The dependences of the permeate flux and separation factor on the linear velocity of the feed mixture are shown in Figure 7.

As can be seen, the permeate fluxes and the separation factor gradually increase with the feed flow velocity, reaching a plateau at 2.4 cm·s^−1^. These dependencies illustrate the disappearance of the diffusion boundary layer upon a change in the hydrodynamic regime in the feed mixture, which leads to a decrease in the concentration polarization effect in the system. Similar trends were obtained in studies where the removal of MTBE from water using highly permeable membranes was investigated [31].

**Figure 8:**
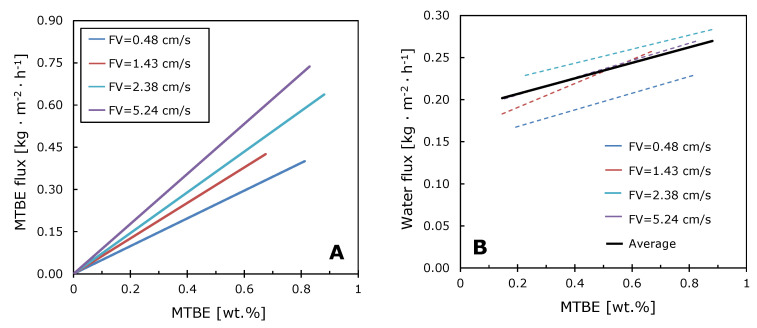
The dependences of MTBE (**A**) and water (**B**) fluxes on MTBE concentration and linear velocity of the feed solution. T = 50 °C. FV–flow velocity.

The dependences of MTBE and water fluxes on the concentration of MTBE at different velocities of the feed solution are more informative (Figure 8A,B). MTBE fluxes rise linearly with the organic component concentration, and the slope of MTBE flux increases with fluid velocity, which indicates a decrease in the concentration polarization effect.

The water flux does not depend on the feed flow velocity and slightly increases with the MTBE concentration. This is due to the swelling of the membrane material with an increase in the organic component concentration. The water concentration is 99+ wt.%. and changes negligibly from the bulk solution to the membrane surface; therefore, concentration polarization does not affect the water flux.

**Figure 9:**
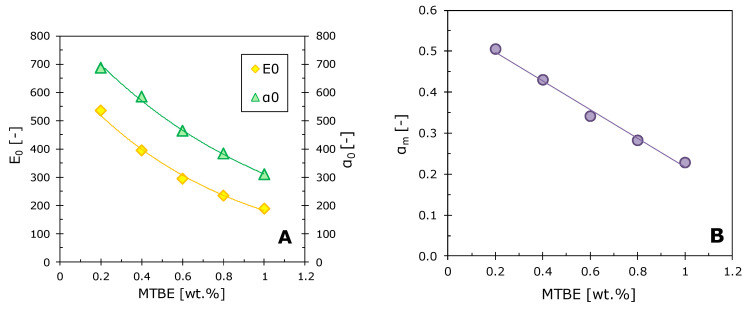
The dependence of the intrinsic enrichment factor E0 and the intrinsic separation factor *α*_0_ (**A**) and the dependence of the membrane selectivity *α_m_* (**B**) on the MTBE concentration in the feed solution. T = 50 °C.

The intrinsic enrichment factor was calculated for the M10/MFFK membrane for the MTBE concentration range 0.2–1.0 wt.%, using the experimental data presented in Figure 8. The calculation was carried out according to the method described in Reference [43]. The intrinsic enrichment factor (*E*_0_) is a value that characterizes the separation properties of the membrane and is independent of the processes occurring in the liquid boundary layer. Knowing the intrinsic enrichment factor, it is easy to calculate the corresponding intrinsic separation factor. The dependences of these two values are shown in Figure 9a. It is evident that enrichment and separation factors decrease with increasing of MTBE concentration in the feed solution. Similar trends were observed in the works [55,60], where the removal of MTBE from water using polysiloxane membranes was studied.

In order to identify the reasons for the drop in the separation factor with an increase of MTBE concentration, the selectivities of the MTBE/water membrane were calculated using Equation (9) and plotted in Figure 9B. A decrease in the separation factor is clearly associated with a decrease in the selectivity of the membrane for the organic component. This phenomenon may result from membrane swelling with an increase in MTBE concentration. A similar effect was observed for polysiloxane membranes when vapors were extracted from gas mixtures [52,61,62].

**Figure 10:**
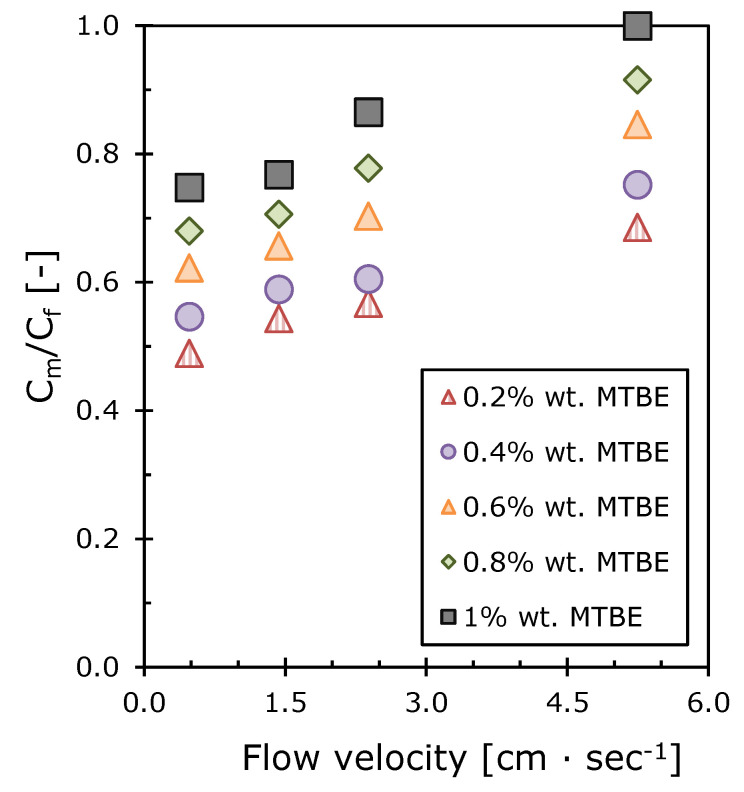
The dependences of the concentration polarization modulus on the feed flow velocity at various MTBE concentrations.

The intrinsic enrichment factor *E*_0_ is a key value needed to calculate the concentration polarization modulus and the boundary layer thickness between the membrane and the feed solution, using Equations (14) and (15) [42]. The calculated values of the concentration polarization modulus continuously increase with the feed flow velocity for all MTBE concentrations (Figure 10). Similar trends were obtained in the previous studies where concentration polarization was analyzed in the process of pervaporation of organochlorine compounds [63] and methyl acetate [43] from a water solution. The concentration polarization modulus increases with the organic composition of the feed mixture (Figure 10), which is consistent with the results of Reference [64].

**Figure 11:**
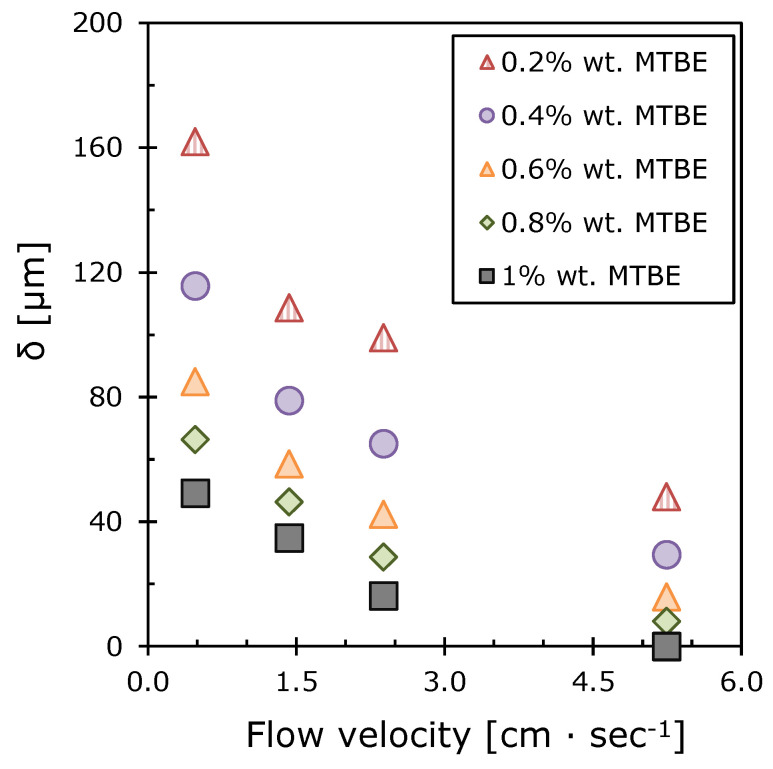
The dependences of the boundary layer thickness on the feed flow velocity at various MTBE concentrations.

Another important indicator of concentration polarization is the boundary layer thickness between the membrane and the bulk liquid. Figure 11 shows the dependences of the boundary layer thickness on the concentration of MTBE in the feed mixture, calculated using (15). As expected, the boundary layer thickness decreases with increasing feed flow velocity [42]. The boundary layer thickness *δ* varies from 0 to 160 μm, which corresponds to the published data (0–300 μm) calculated for the pervaporation of organic components from dilute water solutions [43,65,66]. It decreases with increasing MTBE concentration in the feed mixture which is consistent with the calculated data for the concentration polarization modulus (Figure 10).

In Figure 11 it can be seen that the boundary layer thickness drops sharply when the feed flow velocity rises from 0.5 to 2.0 cm·s^−1^. This indicates a change in the hydrodynamic regime in the membrane module. The boundary layer thickness approaches zero at the feed flow velocity of 5.2 cm·s^−1^ and MTBE concentration 1.0 wt.% This is in good agreement with the value of the concentration polarization modulus approaching 1 and it explains the absence of diffusion difficulties in the liquid phase. However, at lower MTBE concentrations in the solution, the role of concentration polarization is significant for the separation process. It has been shown that this problem for the given concentration range of MTBE can be solved by increasing the flow velocity. For example, extrapolating the concentration polarization modulus graph to 1 for a 0.2 wt.% MTBE solution (Figure 10) indicates the disappearance of the diffusion layer at the feed flow velocity of 13 cm·s^−1^. This method allows optimization of the hydrodynamic regime of pervaporation in order to achieve maximum separation selectivity at minimum cost for the feed solution pumping. It is essential to ensure the required removal rate in one pass and to reduce the cost of the pumping equipment [31].

## 4. Conclusions

The effect of the length of a hydrocarbon side-chain on the transport properties of polyalkylsiloxanes in the pervaporation of MTBE-water solutions has been investigated for the first time. It was shown that the increase in the length of the alkyl side-chain from C1 up to C10 allowed to increase MTBE/water separation factor from 111 (PDMS) to 180 (PDecMS) during the pervaporation of 1 wt.% MTBE solution in water through the dense film (40–50 µm). Due to decrease of the polymer chains flexibility with the increase in the length of the alkyl side-chain (Tg, PDMS = −123 °C, Tg, PDecMS = −68 °C), the overall flux dropped from 0.38 (PDMS) down to 0.12 (PDecMS) kg·m^−2^·h^−1^. It is interesting to notice that the composite membrane with the selective layer made of polydecylmethylsiloxane (~8 µm) on top of a microfiltration support (MFFK membrane) demonstrated an MTBE/water separation factor of 310, which was 72% greater than for the dense film (180). Such a dramatic increase in the separation performance can be attributed to partial penetration of silicone material into the porous structure of the support layer resulted in its restricted swelling. A high separation factor together with overall flux of 0.82 kg·m^−2^·h^−1^ allowed this M10/MFFK composite membrane to outperform the best commercial composite membranes.

Phase transition separation factor and membrane selectivity were calculated in order to assess the role of the membrane in the pervaporation of an MTBE-water solution. It has been proven that an increase in the separation factor with temperature is associated with an increase in the phase transition separation factor. It was found that the high permeability and selectivity of the M10/MFFK membrane used in the pervaporation process leads to a concentration polarization effect in a thin liquid boundary layer. The concentration polarization modulus continuously increases and the boundary layer thickness reduces with the flow rate for all MTBE concentrations. The boundary layer thickness approaches zero at the feed flow velocity of 5.2 cm·s^−1^ for a 1.0 wt.% MTBE solution which means diffusion rate ceases to be a limiting factor of the pervaporation process. However, at lower MTBE concentrations in the solution, the role of concentration polarization is significant for the separation. It has been shown that this problem for the given concentration range of MTBE can be solved by increasing the flow rate. The evaluation of the concentration polarization effect is especially important since we are investigating highly selective membranes that make high MTBE mass transfer coefficients possible. The use of composite membranes based on PDecMS and optimization of separation conditions to minimize concentration polarization is an important step towards the implementation of pervaporation for removal MTBE from wastewater.

## Figures and Tables

**Table 1:** Dense polyalkylmethylsiloxane membranes.

Membrane Symbol	Polymer	Polymer Abbreviation	Modifier	T_g_, °C
M1	polydimethylsiloxane	PDMS	-	−123
M7	polyheptylmethylsiloxane	PHepMS	1-hepten	−99
M8	polyoctylmethylsiloxane	POMS	1-octen	−93
M10	polydecylmethylsiloxane	PDecMS	1-decen	−68

**Table 2:** The physicochemical properties of water, MTBE and polyalkylmethylsiloxanes with a different side-chain (M1, M7, M8 and M10).

Properties of Solvents	Water		MTBE	
Molecular weight [g mol^−1^]	18.01		88.15	
Molecular volume [ml mol^−1^]	18		119	
Boiling point, °C	100.0		55.2	
Kinetic diameter, Å	2.65		6.20	
δ_d_, MPa^1/2^	15.5		14.8	
δ_p_, MPa^1/2^	16.0		4.3	
δ_h_, MPa^1/2^	42.3		5.0	
**Properties of Polymers**	**M1**	**M7**	**M8**	**M10**
δ_d_, MPa^1/2^	16.8	16.8	16.8	16.8
δ_p_, MPa^1/2^	6.0	2.5	2.3	2.0
δ_h_, MPa^1/2^	7.4	4.8	4.6	4.3
**Distance Parameters**	**Water**		**MTBE**	
∆_i-M1_, MPa^1/2^	36.6		3.6	
∆_i-M7_, MPa^1/2^	39.8		2.6	
∆_i-M8_, MPa^1/2^	40.1		2.8	
∆_i-M10_, MPa^1/2^	40.6		3.1	

**Table 3:** Pervaporation characteristics of composite membranes used for the removal of MTBE from water solutions.

Membrane	Temperature [°C]	C_MTBE_, Feed Solution [wt.%]	Overall Flux [kg·m^−2^h^−1^]	Separation Factor	Ref.
M10/MFFK	40	1	0.82	310	This work
PVA/CSP-1	20	1	0.60	240	[54]
Pervap 1060	40	1	0.70	270	[55]
Pervap 1070	40	1	0.30	280	[55]
PEBAX 4033	40	1	0.03	33	[55]
Al2O3-5nm-C6	35	1	0.70	1.1	[33]
ZrO2-5kD-C6	35	1	1.65	56	[33]
TiO2-5kD-C6	35	1	1.95	84	[33]
Al2O3-5nm-C6	35	1	2.50	3	[34]
TiO2-5kD-C6	35	1	2.16	91	[34]

## Supplementary Materials

The following are available online at https://www.mdpi.com/2073-4360/12/6/1213/s1, Figure S1: DSC curves of polyalkylmethylsiloxanes.
