# Supplementary material for: High Selective Composite Polyalkylmethylsiloxane Membranes for Pervaporative Removal of MTBE from Water: Effect of Polymer Side-chain
Source: Polymers (Basel). 2020 May 26;12(6):1213. doi: 10.3390/polym12061213 (PMC7362244; doi:10.3390/polym12061213)
Supplement: Supplementary file 1 [file polymers-12-01213-s001.pdf]

Supplementary Materials to Article

“High selective composite polyalkylmethylsiloxane membranes for pervaporative removal of MTBE from water: Effect of polymer side chain”

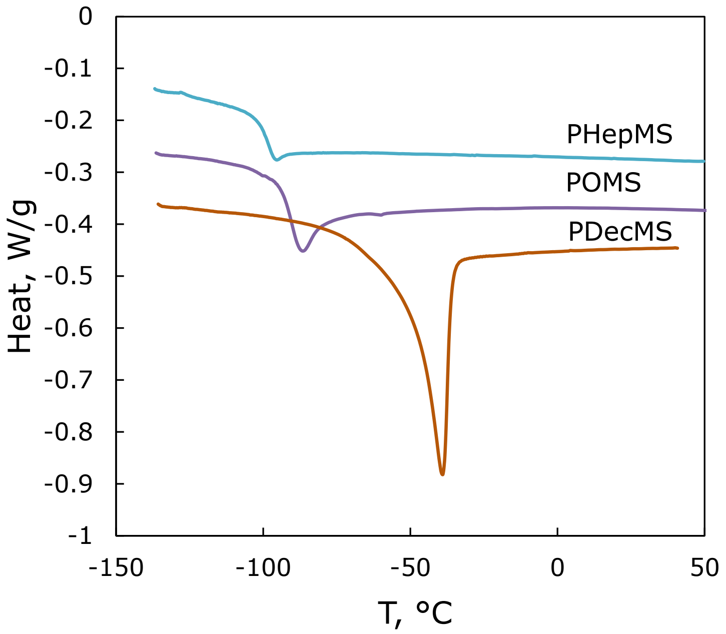

Figure S1: DSC curves of polyalkylmethylsiloxanes.
